# Three-day ceftriaxone versus longer durations of therapy for inpatient treatment of uncomplicated urinary tract infection

**DOI:** 10.1017/ash.2022.317

**Published:** 2022-10-21

**Authors:** Balsam Elajouz, Lisa E. Dumkow, Lacy J. Worden, Kali M. VanLangen, Andrew P. Jameson

**Affiliations:** 1 Department of Pharmacy, Trinity Health Saint Mary’s, Grand Rapids, Michigan; 2 Division of Infectious Diseases, Trinity Health Saint Mary’s, Grand Rapids, Michigan; 3 College of Pharmacy, Ferris State University, Big Rapids, Michigan; 4 College of Human Medicine, Michigan State University, Grand Rapids, Michigan

## Abstract

Current guidelines do not address a recommended duration of parenteral therapy for uncomplicated urinary tract infection (uUTI) treatment in the inpatient setting. We compared a 3-day course of ceftriaxone with longer antibiotic durations for inpatients with a uUTI. Our findings indicate that a 3-day course of ceftriaxone was as efficacious as longer antibiotic courses.

Urinary tract infections are one of the most common indications for antibiotics. Best-practice guidelines recommend 3-day courses of highly bioavailable oral agents, including sulfamethoxazole-trimethoprim (SMZ-TMP) and fluoroquinolones, for outpatient treatment of uncomplicated urinary tract infection (uUTI).^1^ Longer durations (≥5 days) are supported for oral β-lactam agents due to an increased risk for recurrent uUTI with short-course therapy.^2^ Notably, guidelines do not address appropriate durations of therapy for hospitalized patients with uUTI who often receive intravenous (IV) antibiotics and prolonged courses.^3^

Ceftriaxone (CRO) has a suitable spectrum of activity, favorable safety and tolerability profile, and is currently the recommended empiric treatment for complicated UTI in the inpatient setting.^1,4^ Due to its long half-life, IV potency, and similar percentage of urinary secretion compared to first-line oral agents for UTI, a short (ie, 3-day) course of CRO therapy may be efficacious in the treatment of uUTI.^5,6^ We compared a 3-day course of ceftriaxone to longer durations of antibiotic therapy for hospitalized patients with uUTI.

## Methods

### Study design and patient population

**Figure 1.**
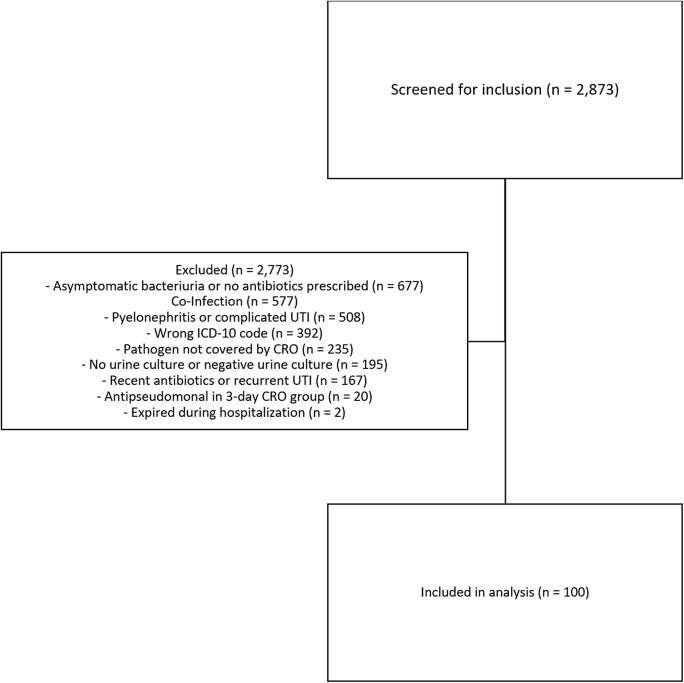
Patients screened for inclusion.

This retrospective cohort study was conducted at a 350-bed community teaching hospital in Grand Rapids, Michigan. The hospital implemented an inpatient antimicrobial stewardship program (ASP) in 2013 and joined the Michigan Hospital Medicine Safety Consortium (HMS) in 2017. The HMS collaborative monitors appropriate treatment of uUTI, focusing on shortest effective durations. As such, the ASP began recommending a 3-day course of CRO for inpatient uUTI in 2019. The study population included hospitalized patients aged ≥18 years receiving antibiotics for documented symptomatic uUTI with a positive urine culture between July 1, 2015, and June 30, 2021. Symptoms meeting criteria for uUTI included at least 1 of the following: dysuria, suprapubic tenderness, urinary frequency, or urinary urgency. The following exclusion criteria were applied: signs or symptoms of systemic infection (eg, fever, flank pain), diagnosis of pyelonephritis or complicated UTI, pregnancy, presence of urinary instrumentation or indwelling device, treated for UTI within the previous 30 days, prior antibiotic use within 7 days, urinary pathogen resistant to the antibiotic regimen prescribed, history of chronic or recurrent UTI, expired during hospitalization, or coinfection. Patients with altered mental status documented as their sole ‘urinary symptom’ were excluded and were considered to have asymptomatic bacteriuria.^7^ In the group who received 3 days of CRO (ie, the 3-day CRO group), patients were also excluded if they had received an empiric dose of another antibacterial agent. Patients who received the longer duration of therapy (ie, longer-DOT group) must have received at least 5 days of any antimicrobial therapy (Fig. 1).

### Data collection and study end points

Following institutional review board approval, patients were identified by a report generated from the electronic medical record using discharge *International Classification of Disease, Tenth Revision* (ICD-10) codes for UTI. Eligible patients were randomized and screened for inclusion via chart review until a convenience sample of 100 patients was met. Patient, treatment, and infection characteristics were collected along with patient outcomes. The primary objective was to compare clinical cure between patients treated with 3 days of CRO versus longer DOT. Clinical cure was defined as resolution of uUTI symptoms at 24 hours following antibiotic completion or improvement to complete antibiotics at home for patients in the longer-DOT group who had not completed antibiotics prior to discharge. Secondary outcomes included hospital length of stay (LOS), 30-day return visit due to UTI, development of *Clostridiodes difficile* within 30 days, and adverse drug events. Return visits were collected through EPIC Care Everywhere software (Epic Systems Corporation, Verona, WI) and were defined as any UTI-related revisit to primary care, emergency department, urgent care, or hospitalization.

### Statistical analysis

Baseline patient characteristics were represented with descriptive statistics. The χ^2^ or Fisher exact test was used to compare nominal data, whereas interval data were compared using the Student *t* test or Mann-Whitney *U* test, based on the distribution of data. Two-tailed, type 1 error of 0.05 was considered statistically significant. All statistical analyses were performed using SPSS version 22 software (IBM, Armonk, NY).

## Results

In total, 100 patients were included: 51 patients in the 3-day CRO group and 49 patients in the longer DOT group. Baseline and microbiology characteristics were similar between groups (Table 1). The most common reason for hospital admission in both groups was uUTI (3-day CRO 33.3% vs longer DOT 24.5%; *P* = .486). In the longer-DOT group, CRO was the most prescribed empiric antibiotic (65.3%) followed by oral cephalexin (20.4%), and 36.7% of patients received IV therapy for their entire course. The median duration of therapy in the longer-DOT group was 6 days (IQR, 5–7), with a maximum treatment duration of 14 days.

**Table 1.** Baseline and Treatment Characteristics

Variable	uUTI Therapy		*P* Value
	3-Day CRO (n=51)	Longer-DOT (n=49)	
Sex, female, no. (%)	46 (90.2)	38 (77.6)	.106
Age, mean y (± SD)	76.5 (13.6)	72.8 (14.2)	.187
Creatinine clearance, mL/min (± SD)	42.7 (28.4)	44.1 (23.8)	.780
Charlson comorbidity index, median (IQR)	5 (4-7)	5 (4-7)	.892
Empiric intravenous therapy, no. (%)	51 (100)	34 (69.4)	<.001
**Empiric antibiotic received, no. (%)**			.003
Amoxicillin	0 (0)	1 (2)	
Amoxicillin/clavulanate	0 (0)	1 (2)	
Ceftriaxone	51 (100)	32 (65.3)	
Cephalexin	0 (0)	10 (20.4)	
Ciprofloxacin	0 (0)	1 (2)	
Piperacillin/tazobactam	0 (0)	1 (2)	
Nitrofurantoin	0 (0)	2 (4.1)	
Sulfamethoxazole/trimethoprim	0 (0)	1 (2)	
Definitive oral therapy, n (%)	0 (0)	31 (63.7)	<.001
**Pathogen, no. (%)**			
*Escherichia coli*	36 (70.6)	35 (71.4)	.926
*Klebsiella* spp	6 (11.8)	8 (16.3)	.511
*Proteus* spp	3 (5.9)	5 (10.2)	.335
*Enterobacter* spp	3 (5.9)	2 (4.1)	.680
*Aerococcus* spp	2 (3.9)	4 (8.2)	.372
*Citrobacter* spp	3 (5.9)	0 (0)	.129
Other	1 (2)	1 (2)	.742

Note. uUTI, uncomplicated urinary tract infection; CRO, ceftriaxone; DOT, days of therapy.

We did not detect a difference in the primary end point between groups because all patients achieved clinical cure (*P* = 1.0). Additionally, we detected no differences in secondary end points between groups, including median hospital LOS, 30-day return visit due to UTI, and *C. difficile* within 30 days of treatment (Table 2). One patient in the longer-DOT group experienced rigors and flushing during ceftriaxone therapy.

**Table 2.** Patient Outcomes

Variable	uUTI Therapy		*P* Value
	3-Day CRO (n=51)	Longer-DOT (n=49)	
Clinical cure, no. (%)	51 (100)	49 (100)	1
Hospital length of stay, median d (IQR)	5 (4–7)	4 (3–6.5)	.48
*C. difficile*, no. (%)	1 (2)	3 (6.1)	.36
**30-day return visit, no. (%)**	7 (13.7)	3 (6.1)	.319
Female	6 (85.7)	2 (66.7)	.284
Male	1 (14.3)	1 (3.3)	1
**Location of return visit, no. (%)**			
Primary care office	3 (5.9)	3 (6.1)	1
Urgent care	0 (0)	0 (0)	1
Emergency department	2 (3.9)	0 (0)	.495
Hospital admission	2 (3.9)	0 (0)	.495
Adverse drug events, no. (%)	0 (0)	1 (2)	.49

Note. uUTI, uncomplicated urinary tract infection; CRO, ceftriaxone; DOT, days of therapy.

## Discussion

Our findings suggest that 3-days of CRO may be as efficacious as longer durations for uUTI in inpatients. In the outpatient setting, a 3-day course of therapy is commonly accepted. Vogel et al^8^ compared 3-day versus 7-day courses of CRO in 183 elderly women with uUTIs.^8^ Bacterial eradication 2 days following treatment was similar between groups (98% vs 93%; *P* = .16), with significantly fewer adverse events in the 3-day group (1.2% vs 2.1%; *P* < .001).^8^ Norrby et al^2^ reviewed 28 trials of women with uUTIs and found that oral β-lactam antibiotics had higher rates of failure with treatment durations of 3 days compared to SMZ-TMP. These researchers proposed that pharmacokinetic properties could account for these differences, owing to the longer plasma half-life of SMZ/TMP and thus, longer amount of time with therapeutic urinary concentrations.^2^ Because the half-life of CRO is similar to that of SMZ-TMP, CRO may have similar efficacy with a 3-day course.

Our findings contribute to filling the current gap in national uUTI treatment guidelines, and these findings align with recent literature supporting short-course therapy.^1,9^ These findings are important for hospital ASPs because uUTI is commonly diagnosed and results in significant over-prescribing as reported by Vaughn et al,^3^ who found that 38.7% of patients with UTI received excessive antibiotic courses at hospital discharge.^3^ Shortening durations of therapy limits antibiotic exposure, reducing resistance and patient harm.

This study had several limitations. Our sample size was small due to stringent exclusion criteria to ensure that only symptomatic patients were included. Notably, our patient population was elderly, with a median Charlson comorbidity index of 5, and these patients may be more frail than patients seeking uUTI treatment in the outpatient setting. Male patients diagnosed with uUTI were included because recent literature has reported the efficacy of short-course therapy for this population.^10^ Additionally, this single-center, retrospective analysis relied on accurate documentation, which may limit external validation. Finally, though our study focused on short-course parenteral therapy, some patients were likely eligible for oral therapy, raising the question of whether full-course parenteral therapy is appropriate for all patients. For those who could transition to oral therapy after receiving CRO initially, the ideal duration of antibiotics is yet to be determined.

In conclusion, a 3-day course of IV CRO is likely an effective treatment strategy for inpatient uUTI and may limit prolonged antibiotic durations. Future study on the shortest effective inpatient uUTI treatment is warranted.
